# Duo Shared Genomic Segment analysis identifies a genome-wide significant risk locus at 18q21.33 in myeloma pedigrees

**DOI:** 10.20517/jtgg.2021.09

**Published:** 2021-05-27

**Authors:** Rosalie Griffin Waller, Michael J. Madsen, John Gardner, Douglas W. Sborov, Nicola J. Camp

**Affiliations:** 1 Huntsman Cancer Institute, Salt Lake City, UT 84112, USA.; 2 University of Utah School of Medicine, Salt Lake City, UT 84112, USA.; 3 Quantitative Health Sciences, Mayo Clinic, Rochester, MN 55905, USA.

**Keywords:** High-risk pedigrees, gene mapping, multiple myeloma, disease susceptibility

## Abstract

**Aim:**

High-risk pedigrees (*HRPs*) are a powerful design to map highly penetrant risk genes. We previously described Shared Genomic Segment (SGS) analysis, a mapping method for single large extended pedigrees that also addresses genetic heterogeneity inherent in complex diseases. SGS identifies shared segregating chromosomal regions that may inherit in only a subset of cases. However, single large pedigrees that are individually powerful (at least 15 meioses between studied cases) are scarce. Here, we expand the SGS strategy to incorporate evidence from two extended HRPs by identifying the same segregating risk locus in both pedigrees and allowing for some relaxation in the size of each HRP.

**Methods:**

Duo-SGS is a procedure to combine single-pedigree SGS evidence. It implements statistically rigorous duo-pedigree thresholding to determine genome-wide significance levels that account for optimization across pedigree pairs. Single-pedigree SGS identifies optimal segments shared by case subsets at each locus across the genome, with nominal significance assessed empirically. Duo-SGS combines the statistical evidence for SGS segments at the same genomic location in two pedigrees using Fisher’s method. One pedigree is paired with all others and the best duo-SGS evidence at each locus across the genome is established. Genome-wide significance thresholds are determined through distribution-fitting and the Theory of Large Deviations. We applied the duoSGS strategy to eleven extended, myeloma HRPs.

**Results:**

We identified one genome-wide significant region at 18q21.33 (0.85 Mb, *P* = 7.3 × 10^−9^) which contains one gene, *CDH20*. Thirteen regions were genome-wide suggestive: 1q42.2, 2p16.1, 3p25.2, 5q21.3, 5q31.1, 6q16.1, 6q26, 7q11.23, 12q24.31, 13q13.3, 18p11.22, 18q22.3 and 19p13.12.

**Conclusion:**

Our results provide novel risk loci with segregating evidence from multiple HRPs and offer compelling targets and specific segment carriers to focus a future search for functional variants involved in inherited risk formyeloma.

## INTRODUCTION

Multiple myeloma (MM) is the second most common adult-onset lymphoid neoplasm and has the worst 5-year survival^[1]^. Inherited germline susceptibility for MM is consistently supported^[2]^: excess MM risk among relatives has been observed in family aggregation^[3,4]^, epidemiologic case-control^[5–9]^, and registry-based^[10,11]^ studies. Disease rarity, short survival, clinical and locus heterogeneity challenge study ascertainment and genetic discovery^[12]^. Genome-wide association studies have identified 23 loci harboring common-risk single nucleotide polymorphisms (SNPs) for MM^[13–19]^. Family-based studies have identified rare germline variants in *ARID1A* and *USP45*^[20]^, *KDM1A*^[21]^, and *DIS3*^[22]^ in exome sequencing. However, considerable missing heritability remains. Additional approaches are needed to aid the detection of the remaining risk loci and genes.

We recently described a novel strategy to map genes involved in complex disease risk using extremely large high-risk pedigrees and allowing for intra-familial heterogeneity, called Shared Genomic Segment (SGS)^[20]^. Cases sharing genomic segments from a common ancestor through 15 meioses or more are unexpected at a genome-wide level^[23]^, and hence a single large high-risk pedigree (HRP) can provide the power to identify novel loci with genome-wide significance^[24]^. Our resource of eleven large myeloma pedigrees included several with 3–4 cases and meioses in the 8–14 range^[20]^. While these remain extremely large families, they may lack sufficient power individually for genome-wide significance. Also, a multi-pedigree strategy is attractive. Evidence for the same risk locus in two extended pedigrees adds confidence to the locus and can build on the power of both. The remaining challenge for any multi-pedigree approach, however, is to adequately address heterogeneity between pedigrees^[25]^.

Here, we expand the SGS method based on combining evidence from pairs of HRPs, while still allowing for intra-familial heterogeneity within each pedigree. In our approach, *duo-SGS*, we fix one pedigree and optimize over all pedigree pairs to balance discovery with multiple testing. Both pedigrees must have a segregating genomic segment at the same risk locus. The method is robust to allelic heterogeneity as different alleles at the same locus may be shared within each pedigree. We apply the duo-SGS approach to eleven MM HRPs to identify novel loci involved in myeloma risk.

## METHODS

### Duo-SGS method

**Figure 1.**
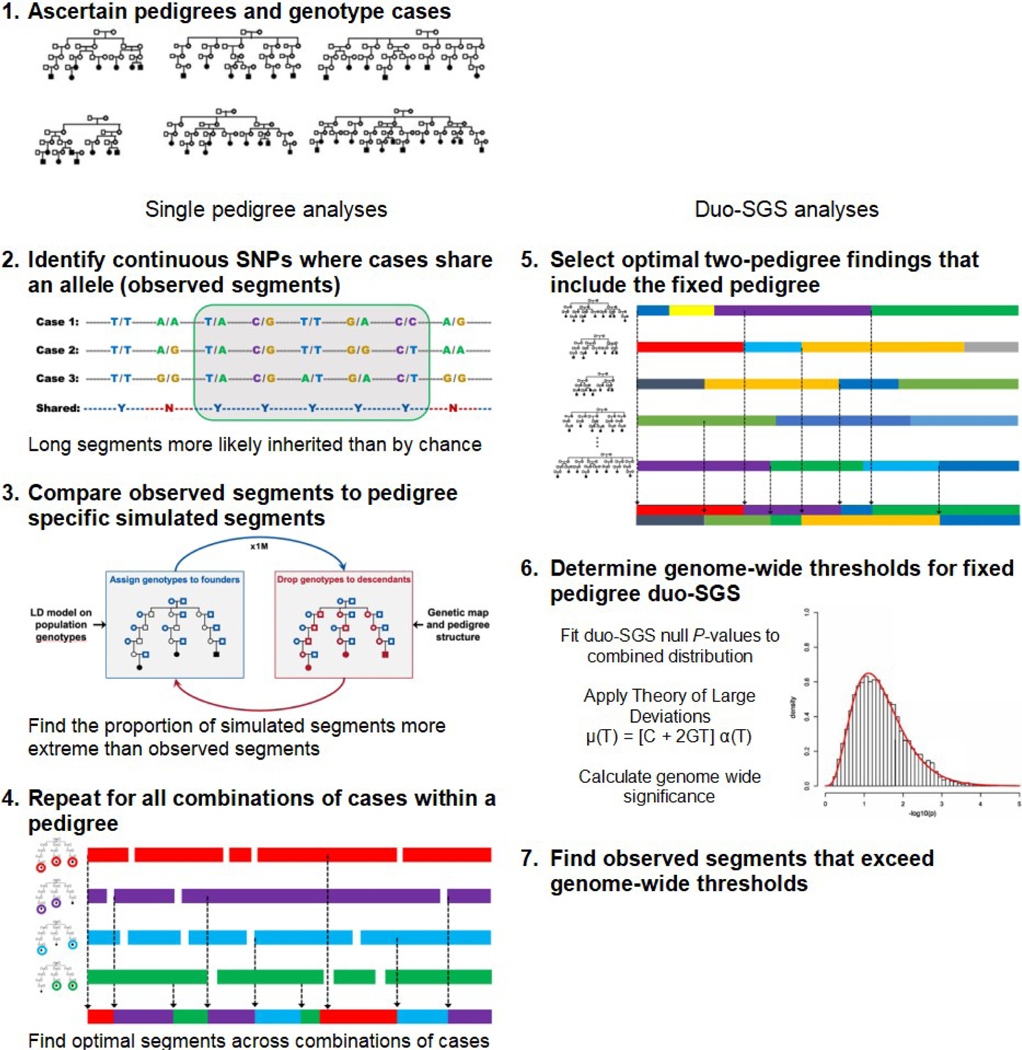
Overview of the duo-SGS method. SGS: Shared genomic segment; SNP: single nucleotide polymorphism.

An overview of the duo-SGS approach can be found in Figure 1. After identifying HRPs and genotyping cases, the observed shared genomic segments in single pedigrees are established and compared between pedigrees, and genome-wide thresholds are determined.

### Observed duo-SGS sharing

The single pedigree SGS approach has been described previously^[20]^. Briefly, the single SGS approach identifies shared observed genomic segments by defining consecutive runs of SNPs that are identity-by-state in a group of cases (Figure 1, Step 2). If the length of an observed segment is significantly longer than it would be by chance, inherited sharing (identity-by-descent) is implied. The nominal significance of each segment is assessed empirically. Expected length sharing under the null hypothesis is generated using a gene-dropping algorithm (Figure 1, Step 3). Chromosomes are assigned to the pedigree founders (those with no parents in the pedigree) randomly and according to a population linkage disequilibrium model. These simulated chromosomes are “dropped” through the pedigree structure using Mendelian inheritance expectations according to a genetic map for recombination. All members of the pedigree receive genotypes under the null hypothesis, and simulated genomic segments from this null configuration are established. These simulations are repeated at least one million times. The empirical *P*-value for an observed segment is the proportion of simulated segments that are identical or encompass the observed segment to the number of simulations. All subsets of at least two cases within a pedigree are assessed for observed segments. Then, at every position across the genome, the best evidence (lowest empirical *P*-value) for an excessive length of sharing is established (Figure 1, Step 4). This process results in a final optimized set of shared segments for a single pedigree. Each optimal segment corresponds to a specific subset of cases and has a nominal empirical *P*-value.

For two pedigrees, the duo-SGS evidence is the combination of the nominal empirical *P* -values for the optimal segments at the same genome position in the two pedigrees. Specifically, the Fisher method to combine *P*-values was used. All possible pedigree pairs could be considered as separate analyses, but there are ${}^{n}C_{2}$ pedigree pairs (ways to select 2 pedigrees from *n* total pedigrees), and hence multiple testing can rapidly become an issue. Alternatively, a single analysis comprising optimization across all pedigree pairs could be considered, but this global approach may cloud individual pedigree-pair findings. To balance these two extremes, we propose a fixed-pedigree duo-SGS strategy (Figure 1, Step 5). The procedure is as follows: (1) fix a pedigree of interest; (2) calculate genome-wide duo-SGS evidence for the fixed pedigree with each of the other pedigrees; and (3) optimize across the duo-SGS findings to identify the most significant duo-SGS result at each point across the genome. The optimized findings over pedigree pairs are the duo-SGS results for the fixed pedigree. In this approach, we identify the best two-pedigree results that include the fixed pedigree. The procedure is then repeated for each pedigree, thus producing duo-SGS results for each pedigree.

### Genome-wide thresholds for duo-SGS

Critical to interpreting the observed duo-SGS results are genome-wide significance duo-SGS thresholds for each pedigree (Figure 1, Step 6). To establish these, we echo the same optimization process in null data. Establishing these thresholds is similar to the calculation described for the single pedigree SGS method^[20]^. Under the reasonable assumption that the vast majority of the genome represents chance sharing (i.e., most of the genome does not contain a disease risk gene) we model the distribution for null sharing on the distribution of the empirical *P*-values for each pedigree. To avoid comparing the findings to themselves or skewing to possible true-positives, the empirical-*P*-values are perturbed, and the distribution-fitting is performed at 1 million simulations. The latter is to avoid inappropriate distribution-fitting to extreme outliers, the few results from the alternate hypothesis if included at their final resolution. To perturb an empirical *P*-value we determine its Wilson score 95% confidence interval (CI) (Equation 1) and randomly sample a value from within it.

Equation 1 $$CI=\frac{1}{1+\frac{z^{2}}{n}}\left(\hat{p}+\frac{z^{2}}{2n}\right)\pm \frac{z}{1+\frac{z^{2}}{n}}\sqrt{\frac{\hat{p}(1-\hat{p})}{n}+\frac{z^{2}}{4n^{2}}}$$

where $\hat{p}$ is the empirical *P*-value, *z* is 1.96 (for the 95%CI), and *n* is the number of simulations (here, 1,000,000). The Wilson interval was selected because it always produces non-negative confidence bounds for the *P*-values. The genome-wide set of perturbed empirical *P*-values for a pedigree are considered the “null” *P*-values for that single pedigree. The duo-SGS procedure (described above) is performed using the single pedigree genome-wide null *P*-values. The result of this process is a set of optimal duo-SGS null *P*-values.

Genome-wide significant and suggestive thresholds are determined following our previously described method for single pedigree SGS^[20]^. Briefly, the null duo-SGS *P*-values are log-transformed and fitted to a gamma distribution. The shape (*k*) and rate (σ) parameters of the fitted distribution are applied using the Theory of Large Deviations to calculate the significance thresholds by solving:

Equation 2 μ(X)=[C+2GX]α(X)

where μ(X) is the genome-wide false positive rate, *C* is the number of chromosomes, α(X) is the probability of exceeding $X∼\chi_{2k}^{2}$, and *G* is the genome length in Morgans^[26]^. The false-positive rate is set to 0.05 for the genome-wide significant threshold and 1.0 for the genome-wide suggestive threshold. After solving for *X*, the threshold, *T* is determined by $T=10^{\frac{-X\sigma }{2}}$. Thresholds are specific to each fixed pedigree to assess their duo-SGS results.

### MM high-risk pedigrees

The statewide Utah Cancer Registry (UCR) has been an NCI-supported Surveillance, Epidemiology, and End Results (SEER) Program registry since its inception in 1966. The UCR was utilized to invite all individuals with myeloma in the state to participate. Peripheral blood was collected for DNA extraction from individuals who completed informed consent.

The Utah Population Database (UPDB) is a unique resource^[27]^. It includes a 16-generation genealogy of approximately 5 million people with at least one event in Utah that is record-linked to the UCR and state vital records. Using the UPDB, ancestors whose descendants have an excess of disease based on internal cancer rates and years at risk can be identified and studied as HRPs. The UPDB was used to identify ancestors whose descendants showed a statistical excess of MM (*P* < 0.05). The expectation was based on internal disease rates based on birth cohort, sex, birthplace (in/outside Utah), and years at risk. The total number of myeloma cases in each HRP identified ranged from 4 to 37 cases. After annotating the pedigrees with those with DNA, 11 pedigrees were identified to contain 3 or 4 myeloma cases with DNA (28 individuals; 8 individuals were in more than one pedigree). In each pedigree, the cases were separated by 8 to 23 meioses.

DNA from the 28 cases was genotyped on the Illumina Omni Express high-density SNP array at the University of Utah. Only high-quality bi-allelic SNPs and individuals with adequate call rates across the genome were included. The PLINK software^[28]^ was used for quality control. SNPs with < 95% call rate across the 28 individuals were removed. After filtering, 678,447 SNPs remained. These SNPs were transformed to match 1000Genomes strand orientation.

Individuals were removed if < 90% of the filtered SNPs are called. One myeloma case had a < 90% call rate and was eliminated from the study. We also checked for sex inconsistency based on the genotypes - all cases passed. PLINK relationship estimates were compared with the UPDB pedigree structures - no issues were found.

The duo-SGS method was applied to the MM pedigrees to identify regions with genome-wide suggestive or significant evidence. Post-hoc, some duo-SGS regions were removed from consideration. Duplicate regions occur when the same pair of pedigrees identify the same region in both their fixed-pedigree results. In these situations, duo-SGS *P*-values are identical, but thresholds vary by which pedigree is fixed, potentially leading to different significance levels. The most significant result was reported, and the lesser removed. If an individual resided in two pedigrees and also shared the region in both pedigrees, the region was removed. If the region spanned a centromere, it was removed. Forty-two suggestive regions were removed as duplicates, involving an overlap individual or at the centromere.

## RESULTS

Duo-SGS findings were identified for each of the eleven MM HRPs. The significance thresholds for each fixed pedigree are in Table 1. One region at 18q21.33 reached genome-wide significance and 13 regions were genome-wide suggestive. Table 2 shows the details of the significant or suggestive regions identified, including the duo-SGS *P*-value, expected rate per genome μ(t), the two pedigrees involved, each segregating shared region in the pedigrees, and the overlapping region.

**Figure 2.**
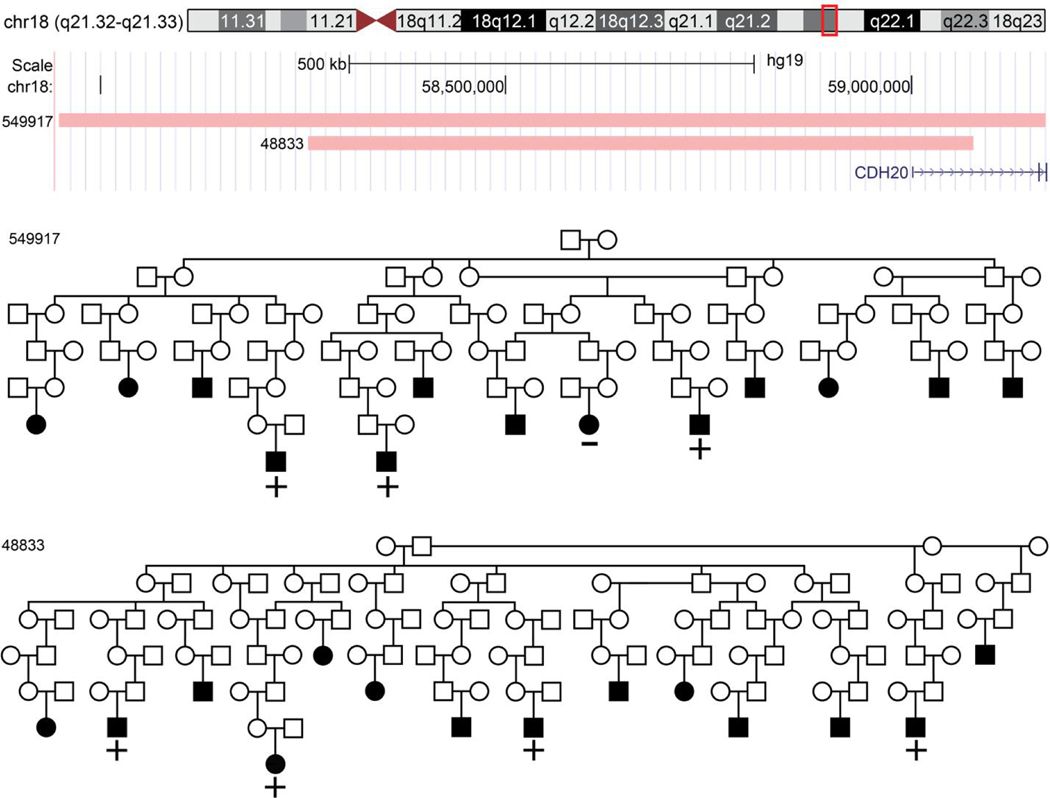
Duo-SGS genome-wide significant region. +/− indicates genotyped cases and SGS carrier status. Squares indicate male and circles female. Filled in shapes have a MM diagnosis. Pedigrees are trimmed to descendants with a MM case. SGS: Shared genomic segment; MM: multiple myeloma.

The genome-wide significant region at 18q21.33 [duo-SGS *P* = 7.3 × 10^−9^, μ(t)] was found in pedigree pair UT-549917/UT-48833. A 1.2 Mb chromosomal segment (chromosome 18 57,945,60259,167,836 bp) segregated to three MM cases separated by 17 meioses in pedigree UT-549917 (single pedigree *P* = 2.8 × 10^−5^). A nested 0.8 Mb chromosomal segment (58,208,260–59,059,262 bp) was observed in four MM cases separated by 23 meioses in pedigree UT-48833 (single pedigree *P* = 1.1 × 10^−5^). The intersecting 0.8 Mb region overlaps one gene: Cadherin 20 (*CDH20*). Figure 2 shows the two regions and the overlap.

Thirteen loci were found with genome-wide suggestive evidence [Table 2]. In four of these loci, several pedigree pairs provide duo-SGS evidence beyond genome-wide suggestive. The locus at 6q16.1 was previously identified as significant in single pedigree SGS in UT-571744, with risk variants in *USP45* implicated^[20]^. Here, we find five pedigree pairs, all including UT-571744, and provide suggestive evidence, including one pair which achieves the second-highest duo-SGS significance in the study [μ(t) = 0.121, *P* = 7.8 × 10^−8^]. The 6q26 region achieves suggestive evidence in four pedigree pairs and harbors the *PARK2* gene. At 5q21.3 four pedigree pairs show suggestive evidence and the locus contains the gene *FBXL17*. The locus at 7q11.23 is also supported by two genome-wide suggestive duo-SGS results. The remaining suggestive loci were supported by one pedigree pair: 1q42.2, 2p16.1, 3p25.2, 5q31.1, 12q24.31, 13q13.3, 18p11.22, 18q22.3 and 19p13.12. Genes in each of the duo-SGS regions are shown in Table 3.

## DISCUSSION

We expanded the shared genomic segment method to identify segregating chromosomal segments with overlapping statistical evidence from two HRPs. The strategy allows for genetic heterogeneity within each pedigree and provides formal significance thresholds for interpretation. The approach circumvents issues of intra-familial heterogeneity that can hinder mapping in large pedigrees. For complex diseases, large HRPs are likely enriched for multiple susceptibility variants^[24]^ and sprinkled with sporadic cases; hence methods that require all cases to share to attain discovery power are not suitable. Here we optimize over subsets within pedigrees and consider pairing with all other pedigrees to provide the flexibility required. The method also specifically defines which pedigrees and cases share evidence at a locus, which is imperative for follow-up sequencing. Additional value may be gained by comparing demographic or clinical characteristics of the sharers in each pedigree to nuance the phenotype which may aid future gene mapping and provide insight into the nature of the mechanism of risk at a locus.

Application of the novel duo-SGS approach to eleven MM HRPs implicated a novel genome-wide significant region at 18q21.33 in MM risk, as well as 13 suggestive regions. Other than 6q16.1, which overlaps with our previous single pedigree SGS study, all loci identified in this study provide novel regions of interest in myeloma. None of the regions overlapped with existing genome-wide association study loci or other prior rare risk variants implicated in MM. A next step would be to investigate the loci for rare and deleterious coding variants or regulatory variants. Pedigree segregation methods can provide statistically compelling regions to concentrate efforts to identify and characterize regulatory risk variants. Also, SGS results can be used as genomic annotations of prior evidence to layer with additional omic information or provide a focused region for interrogating regulatory risk variants.

The literature supports a role of some of the genes found in our duo-SGS regions in MM. The genome-wide significant region at 18q21.33 contained *CDH20*, a gene that plays a role in intracellular adhesion by forming cadherin junctions. Cadherins have been suggested in solid tumor invasion, and metastasis as disruption to cell-cell junctions is a prerequisite^[29]^. Solid tumors co-aggregate in MM families suggesting a shared genetic background^[10]^. At 6q26, several pedigree pairs were genome-wide suggestive, and the overlapping segments fall in *PARK2* which mediates proteasomal degradation. *PARK2* is a tumor suppressor^[30]^ and the gene harbors risk variants for lung cancer^[31]^.

While the duo-SGS approach is useful for analyzing pedigrees smaller than those typically required for the single pedigree SGS approach, a large number of meioses are still required. The HRPs in this study are still substantially larger than those available in most family-based resources (8–23 meioses between sampled cases). Hence the method has practical limitations in other settings. Nonetheless, the interesting regions identified in large pedigrees provide evidence that can be used to narrow the search for risk variants in smaller families as well, as demonstrated in our prior study^[20]^.

As in all family-based genetic studies, our results could be sensitive to inaccurate pedigree structures. However, relationship and ethnicity checks are standard protocols and mitigate the possibility of error. Another limitation to this study is the observational nature. Additional functional studies will be required to describe causation and characterize the mechanisms involved in these loci and myeloma risk.

We have identified several novel loci that segregate in at least two myeloma HRPs. These loci are likely to harbor genes and rare risk variants for MM and are compelling new targets for inherited risk to MM.

In conclusion, we developed a novel strategy for gene mapping in complex traits that uses multiple large high-risk pedigrees. The approach is robust to heterogeneity both within and between pedigrees and formally corrects for multiple testing to allow for statistically rigorous discovery. We applied this strategy to MM, a complex cancer of plasma cells, and identified one novel genome-wide significant locus at 18q21.33 and 13 suggestive loci. Our study offers a new technique for gene mapping and demonstrates its utility to narrow the search for risk variants in complex traits.

## Figures and Tables

**Table 1.** Multiple myeloma high-risk pedigrees and duo-SGS thresholds

Pedigree	Multiple myeloma cases			Duo-SGS thresholds	
	
	Total	Genotyped	Meioses	Significant	Suggestive

260	31	3	16	3.82 × 10^−8^	4.31 × 10^−7^
2122	5	3	18	3.10 × 10^−8^	3.66 × 10^−7^
4823	4	3	13	1.02 × 10^−7^	8.92 × 10^−7^
20245	4	3	13	8.56 × 10^−8^	7.71 × 10^−7^
34955	12	3	16	3.94 × 10^−8^	4.35 × 10^−7^
48833	20	4	23	1.01 × 10^−8^	1.21 × 10^−7^
546699	14	2	11	2.21 × 10^−7^	1.92 × 10^−6^
549917	18	4	21	1.11 × 10^−8^	1.29 × 10^−7^
571744	37	3	20	2.23 × 10^−8^	2.90 × 10^−7^
576834	9	4	16	2.80 × 10^−8^	2.61 × 10^−7^
651626	6	3	13	8.32 × 10^−8^	7.50 × 10^−7^

**Table 2.** Duo-SGS genome-wide significant and suggestive regions

Locus	Duo-SGS				Fixed pedigree					Duo pedigree				
		
	*P*-value	μ(t)	MB	Region	PedID	*P*-value	MB	Region	*N*	Ped ID	*P*-value	MB	Region	*N*

1q42.2	1.08 × 10^−6^	0.464	0.34	233,695,394–234,039,857	546699	1.62 × 10^−3^	1.22	233,288,913–234,508,704	2	549917	3.80 × 10^−5^	0.34	233,695,394–234,039,857	4
2p16.1	2.43 × 10^−7^	0.911	0.11	56,267,182–56,374,910	576834	6.70 × 10^−5^	0.83	56,267,182–57,098,139	4	48833	1.89 × 10^−4^	1.05	55,327,407–56,374,910	3
3p25.2	2.81 × 10^−7^	0.964	1.07	12,006,892–13,075,760	571744	1.80 × 10^−4^	2.03	11,092,264–13,117,999	2	48833	8.20 × 10^−5^	1.07	12,006,892–13,075,760	3
5q21.3	5.36 × 10^−8^	0.354	0.02	107,141,104–107,158,158	549917	4.30 × 10^−4^	0.36	106,800,148–107,158,158	3	48833	6.00 × 10^−6^	0.85	107,141,104–107,994,565	4
	5.27 × 10^−7^	0.630	0.64	107,141,104–107,784,684	651626	4.78 × 10^−3^	0.64	107,140,556–107,784,684	3					
	3.15 × 10^−7^	0.840	0.85	107,141,104–107,994,565	2122	2.78 × 10^−3^	1.69	107,118,121–108,803,319	2					
5q31.1	1.99 × 10^−7^	0.396	0.30	133,295,892–133,591,127	260	1.83 × 10^−4^	0.58	133,011,503–133,591,127	3	549917	5.60 × 10^−5^	1.30	133,295,892–134,597,267	3
6q16.1	8.60 × 10^−8^	0.250	1.71	98,528,404–100,243,033	571744	2.50 × 10^−6^	1.75	98,495,952–100,243,033	3	549917	1.70 × 10^−3^	2.78	98,528,404–101,304,411	2
	7.81 × 10^−8^	0.121	1.23	99,013,740–100,243,033	34955	1.53 × 10^−3^	1.59	99,013,740–100,606,568	2	571744	2.50 × 10^−6^	1.75	98,495,952–100,243,033	3
	3.99 × 10^−7^	0.341	0.98	98,495,952–99,478,304	4823	8.55 × 10^−3^	2.39	97,093,259–99,478,304	2					
	6.03 × 10^−7^	0.752	0.48	98,495,952–98,980,113	651626	1.32 × 10^−2^	0.80	98,177,018–98,980,113	3					
	2.31 × 10^−7^	0.856	0.35	99,893,527–100,243,033	576834	4.81 × 10^−3^	0.40	99,893,527–100,293,531	3					
6q26	1.61 × 10^−7^	0.379	0.48	162,474,893–162,957,575	2122	1.53 × 10^−3^	1.04	162,250,522–163,291,300	2	48833	5.33 × 10^−6^	0.48	162,474,893–162,957,575	4
	1.30 × 10^−6^	0.595	0.23	162,724,247–162,957,575	546699	1.40 × 10^−2^	0.82	162,724,247–163,544,705	2					
	6.20 × 10^−7^	0.752	0.31	162,615,606–162,928,898	20245	6.38 × 10^−3^	0.31	162,615,606–162,928,898	3					
	8.73 × 10^−7^	0.971	0.28	162,474,893–162,752,050	4823	9.17 × 10^−3^	0.30	162,450,471–162,752,050	3					
7q11.23	4.09 × 10^−8^	0.255	1.37	75,441,794–76,813,298	549917	2.90 × 10^−5^	1.66	75,441,794–77,098,772	3	48833	6.70 × 10^−5^	1.59	75,222,070–76,813,298	3
	1.50 × 10^−6^	0.720	1.66	75,442,855–77,098,772	546699	3.00 × 10^−3^	2.22	75,442,855–77,663,486	2	549917	2.90 × 10^−5^	1.66	75,441,794–77,098,772	3
12q24.31	4.50 × 10^−7^	0.494	0.11	121,438,844–121,547,455	20245	9.34 × 10^−4^	1.16	120,388,858–121,547,455	3	571744	2.60 × 10^−5^	1.59	121,438,844–123,030,788	3
13q13.3	2.66 × 10^−7^	0.689	0.13	36,297,517–36,424,698	2122	3.60 × 10^−5^	1.12	35,308,299–36,424,698	3	48833	3.87 × 10^−4^	0.24	36,297,517–36,537,635	4
18p11.22	6.48 × 10^−7^	0.799	0.17	9,693,654–9,866,696	20245	2.90 × 10^−3^	0.19	9,693,654–9,878,670	3	571744	1.23 × 10^−5^	0.58	9,285,824–9,866,696	3
18q21.33	7.30 × 10^−9^	0.029	0.85	58,208,260–59,059,262	549917	2.80 × 10^−5^	1.22	57,945,602–59,167,836	3	48833	1.14×10^−5^	0.85	58,208,260–59,059,262	4
18q22.3	2.72 × 10^−7^	0.569	0.20	68,834,971–69,039,896	34955	2.80 × 10^−4^	0.68	68,357,156–69,039,896	3	48833	5.10 × 10^−5^	0.43	68,834,971–69,260,566	4
19 pi 3.12	9.82 × 10^−7^	0.406	0.44	15,773,427–16,217,107	546699	2.64 × 10^−3^	1.32	15,773,427–17,090,612	2	571744	2.10 × 10^−5^	0.57	15,648,456–16,217,107	3

Genomic coordinates in GRCh37. μ(t) - duo-SGS significance threshold. MB: megabases; N: number of cases sharing the SGS region.

**Table 3.** Protein coding genes in duo-SGS regions by locus

Locus	Gene name	Start	End

1q42.2	*KCNK1*	233,749,750	233,808,258
3p25.2	*TIMP4*	12,194,551	12,200,851
	*PPARG*	12,328,867	12,475,855
	*TSEN2*	12,525,931	12,581,122
	*C3orf83*	12,556,433	12,602,558
	*MKRN2*	12,598,513	12,625,212
	*RAF1*	12,625,100	12,705,725
	*TMEM40*	12,775,024	12,810,956
	*CAND2*	12,837,971	12,913,415
	*RPL32*	12,875,984	12,883,087
	*IQSEC1*	12,938,719	13,114,617
5q21.3	*FBXL17*	107,194,736	107,717,799
5q31.1	*C5orf15*	133,291,201	133,304,478
	*VDAC1*	133,307,606	133,340,824
	*TCF7*	133,450,402	133,487,556
	*SKP1*	133,484,633	133,512,729
	*CTD-2410N18.5*	133,502,861	133,561,762
	*PPP2CA*	133,530,025	133,561,833
	*CDKL3*	133,541,305	133,706,738
6q16.1	*POU3F2*	99,282,580	99,286,660
	*FBXL4*	99,316,420	99,395,849
	*FAXC*	99,719,045	99,797,938
	*COQ3*	99,817,276	99,842,080
	*PNISR*	99,845,927	99,873,207
	*USP45*	99,880,190	99,969,604
	*CCNC*	99,990,256	100,016,849
	*PRDM13*	100,054,606	100,063,454
6q26	*PARK2*	161,768,452	163,148,803
7q11.23	*CCL24*	75,440,983	75,452,674
	*RHBDD2*	75,471,920	75,518,244
	*POR*	75,528,518	75,616,173
	*STYXL1*	75,625,656	75,677,322
	*MDH2*	75,677,369	75,696,826
	*SRRM3*	75,831,216	75,916,605
	*HSPB1*	75,931,861	75,933,612
	*YWHAG*	75,956,116	75,988,348
	*SRCRB4D*	76,018,651	76,039,012
	*ZP3*	76,026,835	76,071,388
	*DTX2*	76,090,993	76,135,312
	*UPK3B*	76,139,745	76,648,340
	*POMZP3*	76,239,303	76,256,578
	*CCDC146*	76,751,751	76,958,850
	*FGL2*	76,822,688	76,829,143
	*GSAP*	76,940,068	77,045,717
12q24.31	*HNF1A*	121,416,346	121,440,315
	*C12orf43*	121,440,225	121,454,305
	*OASL*	121,458,095	121,477,045
13q13.3	*DCLK1*	36,345,478	36,705,443
18p11.22	*RAB31*	9,708,162	9,862,548
18q21.33	*CDH20*	59,000,815	59,223,006
19p13.12	*CYP4F3*	15,751,707	15,773,635
	*CYP4F12*	15,783,567	15,807,984
	*OR10H2*	15,838,834	15,839,862
	*OR10H3*	15,852,203	15,853,153
	*OR10H5*	15,904,761	15,905,892
	*OR10H1*	15,917,817	15,918,936
	*CYP4F2*	15,988,833	16,008,930
	*CYP4F11*	16,023,177	16,045,677
	*OR10H4*	16,059,818	16,060,768
	*TPM4*	16,177,831	16,213,813

Genomic coordinates in GRCh37.
